# Investigating the Prevalence and Predictors of Apathy among the Canadian Long-Term Care Residents: A Secondary Data Analysis

**DOI:** 10.1177/08445621241276613

**Published:** 2024-08-28

**Authors:** Aderonke Agboji, Shannon Freeman, Davina Banner, Joshua Armstrong, Melinda Martin-Khan

**Affiliations:** 1 Department of Nursing, 6727 University of Northern British Columbia, University way, Prince George, British Columbia, Canada; 2 228440 Alzheimer Society of Canada, Toronto, Ontario, Canada; 3 3286 Exeter Medical School, Faculty of Health and Life Sciences, University of Exeter, Exeter, Devon, UK

**Keywords:** Apathy, long-term care facilities, biopsychosocial model, InterRAI, MDS 2.0, Canada, healthcare system

## Abstract

**Background:**

In long-term care facilities (LTCF), apathy is a prevalent issue, leading to cognitive decline, functional impairment, and increased mortality risk. Despite its significance, apathy often remains underrecognized and undermanaged in these settings. Recognizing and addressing the predictors of apathy is critical for early intervention and improved care outcomes.

**Purpose:**

This study aims to assess the prevalence of apathy and identify its associated risk factors among newly admitted residents in the Canadian LTCF, using the InterRAI Minimum Data Set (MDS 2.0).

**Methods:**

We conducted a cross-sectional analysis of MDS 2.0 admission assessment data between 2015 and 2019, covering 157,596 residents across six Canadian provinces and one territory. Apathy was measured using the Apathy Index of the MDS 2.0, with the biopsychosocial model guiding the analysis.

**Results:**

The prevalence of apathy was 12.5% (19,758 individuals). The most significant predictors include cognitive impairments, specific age groups, hearing impairments, vision impairments, facility size and location.

**Conclusions:**

The findings of this study underscore the need for tailored strategies in LTCF to address apathy, considering individual, institutional, and regional variations. Emphasis on environmental and personal factors is crucial in the management and prevention of apathy in these settings.

## Background & purpose

In Canada, as in many developed countries, long-term care facilities (LTCF) are vital for supporting individuals with complex health needs that exceed the scope of community-based care (Freeman et al., 2017). LTCF, also known as nursing homes or personal care homes, cater to those who require specialized care due to chronic physical, mental, or other disabilities, which is not typically available through home care or retirement services (Canadian Institute for Health Information [CIHI], 2013). The significance of LTCF in the healthcare system has been growing; in 2013, there were 2,036 LTCF across Canada, with a projection that by 2041, the number of residents requiring these facilities will increase to 320,000. However, by 2020, the number of residents in these facilities had reached 474,000, surpassing the forecasted needs (Statistics Canada, 2022).

Apathy is a complex and multidimensional construct (Marin, 1990; Marin & Wilkosz, 2005; Robert et al., 2018), that is increasingly observed among those in LTCF (Gerritsen et al., 2005; Jao et al., 2018; Roth et al., 2007; Tang et al., 2018). Cognitively, it manifests as diminished intellectual engagement such as a lack of curiosity or motivation to pursue knowledge or engage in problem-solving activities (Le Heron et al., 2018; Robert et al., 2002). Behaviorally, it is characterized by a reduction in goal-directed actions including a decrease in initiated activities, a decline in participation in previously enjoyed activities, or a lack of response to motivational cues (Marin, 1991). Emotionally, apathy leads to a blunted affective response or emotional indifference (Starkstein & Leentjens, 2008). Socially, it precipitates a withdrawal from social interactions and activities (Sockeel et al., 2006). Thus, individuals with apathy may manifest one or more symptoms depending on the domains implicated (Marin, 1990; Levy & Dubois, 2006; Robert et al., 2018). It is noteworthy that various terms are used to describe apathy in clinical settings, including abulia, negative symptoms, avolition, amotivation, anhedonia and boredom (Thant & Yager, 2019). However, these terms are distinguishable from apathy (Barch et al., 2016). For example, anhedonia is characterized by a diminished capacity to enjoy activities that are usually pleasurable, often appearing as a symptom of depression (Ang et al., 2018; Serretti, 2023). Apathy, in contrast, is marked by a broad disinterest or lack of motivation, without necessarily affecting emotional responses related to pleasure (Fahed & Steffens, 2021).

The prevalence of apathy among LTCF populations has been variably reported in literature, with estimates ranging significantly due to differences in assessment methods and population. For instance, using the Neuropsychiatric Inventory-Nursing Home Version (NPI-NH) to measure apathy, a study involving LTCF residents with Alzheimer's disease (AD) and other dementias observed that 84.1% of residents had apathy (Wood et al., 2000). An observational study which utilized the NH-version of the Apathy Evaluation Scale (AES10) to assess apathy in a sample of LTCF residents with stroke demonstrated that apathy was found in 28% of the individual within this population (van Almenkerk et al., 2015). A comprehensive review found that the frequency of apathy was 69% among 162 residents admitted to LTCF (Starkstein et al., 2006). In other systematic reviews and meta-analytic studies, it has been reported that 50% of the residents in LTCF have apathy (Leung et al., 2021; Zhao et al., 2016).

Research has consistently shown that apathy contributes to a range of adverse outcomes among LTCF residents, including accelerated cognitive decline, increased dependency in daily activities, and a higher risk of mortality (Aguera-Ortiz et al., 2015; Ayers et al., 2017; Lavretsky et al., 2015; Nijsten et al., 2017; van Reekum et al., 2005). These outcomes not only affect the individuals but also pose challenges for caregivers (formal and informal) and the healthcare system (Jao et al., 2019; Wong et al., 2020). The burden of apathy on caregivers is particularly noteworthy, as it often leads to increased caregiver stress and burnout (Wong et al., 2020). The Canadian healthcare system, with its diverse population and unique healthcare policies (Martin et al., 2018), provides a distinct context for examining apathy.

Despite the significance of apathy among LTCF residents, it is rarely diagnosed or specifically addressed (Nijsten et al., 2023). This oversight can be attributed to several factors. Firstly, there is a lack of standardized, validated tools for apathy assessment and diagnosis among the LTCF population (Agboji et al., 2024; Clarke et al., 2011; Volicer et al., 2013; Miller et al., 2021; Tay et al., 2021). Secondly, symptoms of apathy are often mistakenly attributed to other neuropsychiatric conditions like depression, leading to misdiagnosis and inappropriate management (Jao et al., 2019; Leone et al., 2013). Thirdly, prior research has primarily focused on apathy as a secondary symptom of neurodegenerative diseases including Alzheimer's and Parkinson's (Manera et al., 2020; Robert et al., 2009, 2018; Jao et al., 2019). However, recent studies suggest that apathy can exist independently (Lopez et al., 2019; Starkstein et al., 2005).

Existing literature suggests that predictors of apathy in the LTCF included a range of demographic, psychological, and social factors (Ang et al., 2018; Cummings et al., 2015; Drye et al., 2013; Robert et al., 2018; Yuen et al., 2014). Variables such as age, cognitive impairment, depression, and physical health status have been implicated, but findings are not consistent across studies. For example, in a study by Starkstein et al. (2009), it was found that older age was a risk factor for apathy, whereas, in other studies, it was reported that younger age (age below 65 years) was a predictor of apathy (Appelhof et al., 2019; Jao et al., 2018). By contrast, Holtta et al. (2012) found no differences in age among those who have apathy. With regards to sex, some studies showed that being male was a risk factor (Jao et al., 2020; Vilalta-Franch et al., 2013), while other studies found no relationship between sex and apathy (Clarke et al., 2008; Proitsi et al., 2011; Starkstein et al., 2006). These inconsistencies underscore the need for more targeted research using standardized measures.

To date, a comprehensive study examining the factors that may increase the risks of apathy in the LTCF context is sparse. The purpose of this current study was to examine the prevalence and predictors of apathy among persons living in the Canadian LTCF using the Apathy Index of the MDS 2.0, a standardized tool approved for use in LTCF across Canada.

## Methods and procedures

### Study design

This retrospective cross-sectional study analyzed de-identified data from residents in LTCF, using the MDS 2.0 from the Continuing Care Reporting System (CCRS) database of the Canadian Institute for Health Information (CIHI).

### Setting and sample

We included residents from the Canadian LTCF across six provinces (Alberta, British Columbia, Manitoba, Newfoundland and Labrador, Ontario, and Saskatchewan) and one territory (Yukon) who participated in the admission assessments between 2015 and 2019. Exclusions were residents in comatose states or with missing relevant information.

### Data collection

As aforementioned, the MDS 2.0 is a mandated assessment tool used in most Canadian LTCF. It provides comprehensive personal-level information about residents that clinicians may use to inform their decision-making when developing a care plan that reflects the individual's needs, preferences, and strengths (Hirdes et al., 2008). The assessments were completed by trained healthcare staff within 14 days of admission (admission assessment) and quarterly thereafter, involving multiple information sources, including observations and consultations with various healthcare professionals (Hirdes et al., 2008). Built into the MDS 2.0 are the clinical assessment protocols (CAPs), also referred to as outcome scales. The CAPs are used to identify residents who can improve with appropriate care or are at risk of adverse outcomes (Hirdes et al., 2008). This study incorporated several key scales including Cognitive Performance Scale (CPS), which monitors changes in cognitive status (Hirdes et al., 2008); Activity of Daily Living Self-Performance Hierarchy Scale (ADL-hierarchy), offering insight into the progression of residents’ disabilities through ADL performance analysis (Hirdes et al., 2008); the Pressure Ulcer Risk Scale (PURS), aimed at identifying the risk of developing pressure ulcers (Poss et al., 2008); Change in Health, End-Stage Disease, and Signs and Symptoms Scale (CHESS), which evaluates the resident's health instability and aids in predicting mortality (Morris et al., 2012); the Index of Social Engagement (ISE) measures residents’ sense of initiatives and social involvement, and consists of six items, including at ease interacting with others, doing planned or structured activities, doing self-initiated activities; establishes own goals; pursues involvement in the facility's life, and accepting invitations into most groups’ activities (Morris et al., 1999); and the Depression Rating Scale (DRS) measuring mood and behavior (Burrows et al., 2000).

### Apathy measures

Apathy was measured using two items, termed the Apathy Index of the MDS 2.0, assessing withdrawal from activities and reduced social interactions in line with the framework for apathy assessment proposed by Volicer et al. (2013). These two items were rated on a 3-point Likert scale by the CCRS as follows: 0 = Indicator not exhibited in last 30 days; 1 = Indicator of this type exhibited up to 5 days a week; 2 = Indicator of this type exhibited daily or almost daily (6, 7 days a week). We converted these two items into a dichotomous variable. Thus, a score of 0 indicates non-apathetic and 1 is apathetic. Studies have reported a Cronbach's Alpha coefficient of 0.75–0.89 for this measure indicating a high level of internal consistency (Gerritsen et al., 2008; Stones et al., 2006; Volicer et al., 2013). Additionally, these two items are core symptoms of apathy included in the screening questions of the Neuropsychiatric Inventory (NPI) and have demonstrated a high degree of internal consistency (Cummings et al., 1994). High internal consistency indicates that the items on the apathy index are measuring the same underlying concept of apathy consistently across different assessments.

### Data analysis

Data analysis was conducted using SAS version 9.4. (SAS Institute, Cary, NC, USA, 2013). Descriptive statistics described participant characteristics, and simple logistic regression identified apathy predictors. Prevalence was defined as the percentage of LTCF residents with an apathy score of 1. Statistical significance was set at an alpha level of p < .05.

### Theoretical framework

Considering the interplay of biological, psychological, and social influences on apathy, the biopsychosocial model served as the theoretical foundation for this study (Bolton & Gillett, 2019; Ghaemi, 2009). Variables were classified accordingly: biological (age, sex, weight, and sensory impairment), psychological (cognitive performance scores), and social (marital status, language, facility size, and facility province) factors.

## Results

### Sample characteristics

Table 1 presents the sociodemographic characteristics of the study participants. Of the 157,596 residents included in the sample, over half of the population were female (63.5%), 51.1% were aged 85 years or older, 66.6% did not have a partner or spouse (never married, widowed, divorced, or separated), 16% lived alone before admission, 84.9% were English speakers, over two-thirds lived in large LTCF (over 100 beds-Morris et al., 2012) (69.9%), and approximately one quarter entered the facilities from inpatient acute care services (27.9%).

**Table 1.** Sociodemographic characteristics by status of apathy of residents newly admitted to the Canadian LTCF between 2015 and 2019 (N = 157,596).

Variables	Total Population 100% (N = 157,596)	Apathetic 12.5% (n = 19,758)	Non-apathetic 87.6% (n = 137,838)
**Age at admission (years)**			
less than 65	6.04 (9,519)	14.22 (1,354)	85.78 (8,165)
65–74	11.95 (18,840)	13.90 (2,619)	86.10 (16,221)
75–84	30.95 (48,782)	12.73 (6,209)	87.27 (42,573)
85 and above	51.05 (80,455)	11.90 (9,576)	88.10 (70,879)
**Sex**			
Female	63.46 (100, 009)	12.05 (12,052)	87.95 (87,957)
Male	36.46 (57,452)	13.39 (7,690)	86.61 (49,762)
Other	0.09 (135)	11.85 (16)	88.15 (119)
**Marital status**			
Married	29.87 (40,598)	11.87 (4,818)	88.13 (35,780)
Never married/widowed/ separated/divorced	66.61 (90,530)	11.70 (10,590)	88.30 (79,940)
Not specified	3.51 (4,774)	13.57 (648)	86.43 (4,126)
**Lived alone before entry to LTCF**			
No	84 (124,118)	12.34 (15,322)	87.66 (108,796)
Yes	16 (23,648)	12.50 (2,957)	87.50 (20,691)
**Language**			
English	84.85 (133,718)	13.06 (17,462)	86.94 (116,256)
French	2.23 (3,509)	11.97 (420)	88.03 (3,089)
Other	12.92 (20,369)	9.21 (1,876)	90.79 (18,493)
**Facility size**			
Large (100 + beds)	69.78 (109,965)	11.38 (12,513)	88.62 (97,452)
Medium (30–99 beds)	28.00 (44,134)	14.87 (6,561)	85.13 (37,573)
Small (1–29 beds)	2.22 (3,497)	19.56 (684)	80.44 (2,813)
**Entry Service Type**			
Ambulatory health Service	0.63 (991)	13.22 (131)	86.78 (860)
Inpatient Acute Care Service	27.94 (44,031)	14.96 (6,586)	85.04 (37,445)
Inpatient Rehabilitation Service	1.55 (2,450)	11.67 (286)	88.33 (2,164)
Inpatient Continuing Care Service	6.19 (9,758)	13.12 (1,280)	86.88 (8,478)
Residential Care Service	15.11 (23,808)	14.54 (3,461)	85.46 (20,346)
Inpatient Psychiatric Service	1.15 (1,818)	20.96 (381)	79.04 (1,437)
Other/Unclassified service	0.84 (1,323)	13.38 (177)	86.62 (1,146)
Inpatient Rehabilitation Service-Specialized	0.37 (589)	12.05 (71)	87.95 (518)
Home Care Service	9.78 (15,415)	11.14 (1,717)	88.86 (13,698)
Residential Care Service-board and care	15.35 (24,192)	10.25 (2,479)	89.75 (21,713)
Private Home-no home care	21.08 (33,221)	9.60 (3,189)	90.40 (30,032)
**Cognitive Performance Scale (CPS)**			
Intact (score = 0)	9.39 (14,796)	7.98 (1,181)	92.02 (13,615)
Borderline Intact (score = 1)	12.30 (19,839)	11.59 (2,247)	88.41 (17,142)
Mild impairment (score = 2)	22.11 (34,837)	10.80 (3,762)	89.20 (31,075)
Moderate impairment (score = 3)	36.88 (58,120)	13.01 (7,560)	86.99 (50,560)
Moderate severe impairment (score = 4)	7.98 (12,583)	14.11 (1,775)	85.89 (10,808)
Severe impairment (score = 5)	8.84 (13,935)	18.43 (2,568)	81.57 (11,367)
Very severe impairment (score = 6)	2.50 (3,936)	16.90 (665)	83.10 (3,271)
**Activity of daily living self performance hierarchy scale (ADL-hierarchy)**			
Low levels of decline (score = 0–1)	11.80 (18,601)	11.84 (2,202)	88.16 (16,399)
Moderate levels of decline (score = 2–3)	48.41 (76,298)	12.12 (9,248)	87.88 (67,050)
High levels of decline (score = 4–6)	39.78 (62,697)	13.25 (8,308)	86.75 (54,389)
**Pressure ulcer risk (PURS)**			
Low risk (score = 0–2)	71.99 (113,447)	12 (13,616)	88 (99,831)
Moderate risk (score = 3–5)	27.45 (43,264)	13.82 (5,979)	86.16 (37,285)
High risk (score = 6–8)	0.56 (885)	18.42 (163)	81.58 (722)
**Change in Health, End Stage Disease and Signs and Symptoms (CHESS)**			
Low levels of medical complexity (score = 0–1)	84.47 (133,115)	11.26 (14,987)	88.74 (118,128)
Moderate levels of medical complexity (score = 2–3)	14.79 (23,303)	18.77 (4,374)	81.23 (18,929)
High levels of medical complexity (score = 4–5)	0.75 (1,178)	33.70 (397)	66.30 (781)
**Index of Social engagement (ISE)**			
Low levels of engagement (score = 0–1)	20.79 (32,771)	23.33 (7,644)	76.67(25,127)
Moderate levels of engagement (score = 2–3)	40.66 (64,084)	12.20 (7,817)	87.80 (56,267)
High levels of engagement (score = 4–6)	38.54 (60,741)	7.07 (4,297)	92.93 (56,444)
**Depression Rating Scale (DRS)**			
Low levels of depressive symptoms (score = 0–2)	79.64 (125,509)	9.58 (12,020)	90.42 (113,489)
Moderate levels of depressive symptoms (score = 3–5)	15.61 (24,597)	21.29 (5,237)	78.71 (19,360)
High levels of depressive symptoms (score = 6–14)	4.75 (7,490)	33.39 (2,501)	66.61 (4,989)
**Antidepressant use**			
No	54.66 (86, 136)	11.30 (9,732)	88.70 (76,404)
Yes	45.34 (71,460)	14.03 (10,026)	85.97 (61,434)

**Figure 1.**
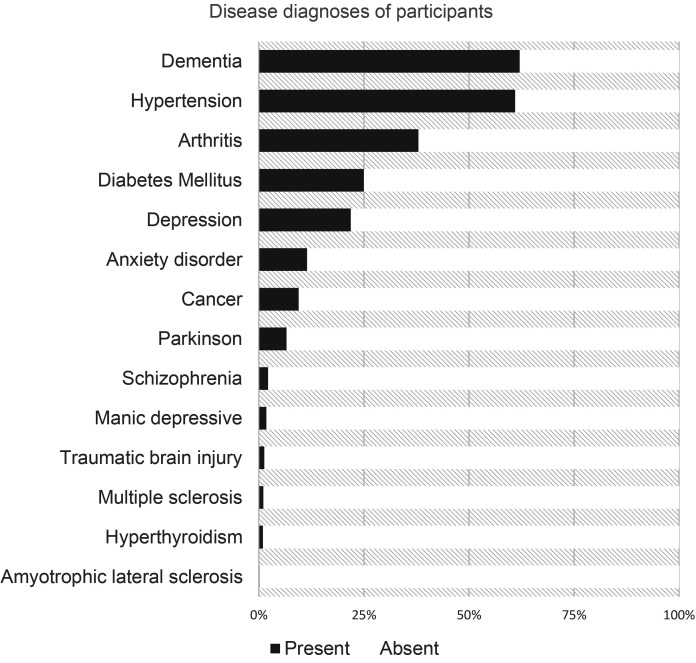
Disease diagnoses of residents newly admitted to the Canadian LTCF between 2015 and 2019 (N = 157,596).

Figure 1 shows the diagnosis of the disease in the study population. Over half of the population (62.1%) was diagnosed with dementia. Hypertension was also prevalent, affecting 61% of the population. A small percentage of residents were diagnosed with amyotrophic lateral sclerosis (0.2%), multiple sclerosis (1.1%), Parkinson's disease (6.6%), manic depressive disorder (1.8%), schizophrenia (2.2%), and traumatic brain injury (1.3%). Additionally, less than a quarter of the population had anxiety disorder (11.5%) and depression (24.1%).

### Prevalence of apathy

**Figure 2.**
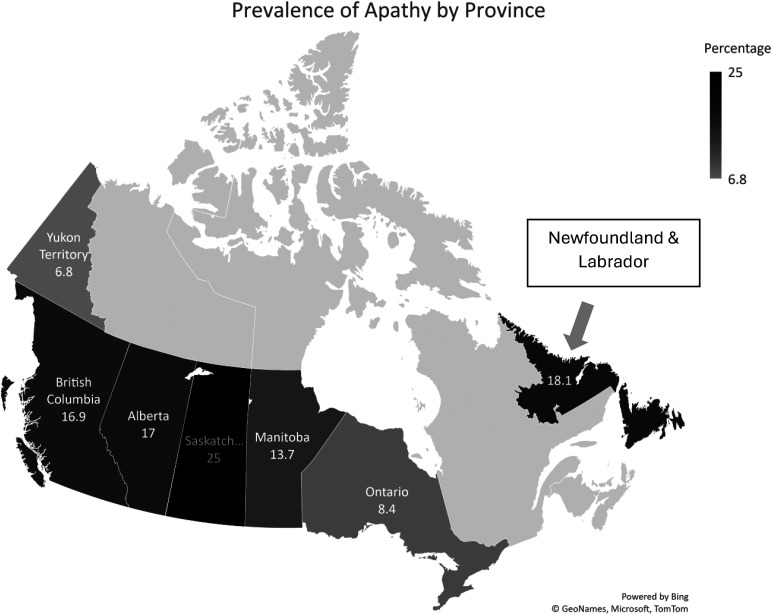
Prevalence of apathy among the newly admitted Canadian LTCF residents by province.

The prevalence of apathy among Canadian LTCF residents, as measured by the Apathy Index of the MDS 2.0 was 12.5% (Table 1). The prevalence of apathy was higher among residents who were under 65 years of age (14.2%) than among those aged 85 years and above (11.9%). Apathy was nearly twice as prevalent among residents of small LTCF compared to those residing in large LTCF (19.6% vs. 11.3%). Additionally, geographical region influenced the prevalence of apathy; it ranged from 6.8% in Yukon to 25.6% in Saskatchewan (Figure 2). Apathy was reported by the highest proportion of individuals (21%) entering LTCF via inpatient psychiatric services, as opposed to individuals who entered the facilities via private homes and were not receiving home care (9.6%). Apathy was more frequent among English speakers (13.1%) compared to French speakers (11.8%) and speakers of other languages (9.2%). No significant differences were found between those who lived alone before admission to LTCF and those who did not.

Furthermore, apathy was more prevalent among residents who had a high score (4+, severe impairment) on various outcome scales than among those who had a low score (0–2, low impairment) (Table 1), including the ADLS-hierarchy (13.3% vs. 11.4%), PURS (18.4% vs 12%), CPS (16.9% vs 10%), DRS (33.4% vs. 9.6%) and CHESS scales (37.7% vs. 11.3%). In addition, the proportion of residents who had a low score on the ISE (0–1, low engagement) and exhibited apathy was higher in comparison with those who had a high score (4+, high engagement) on this item (23.3% vs. 7.1%). Apathy was more prevalent among residents who were using anti-depressants compared to those who did not (14% vs. 11.3%).

### Predictors of apathy

**Figure 3.**
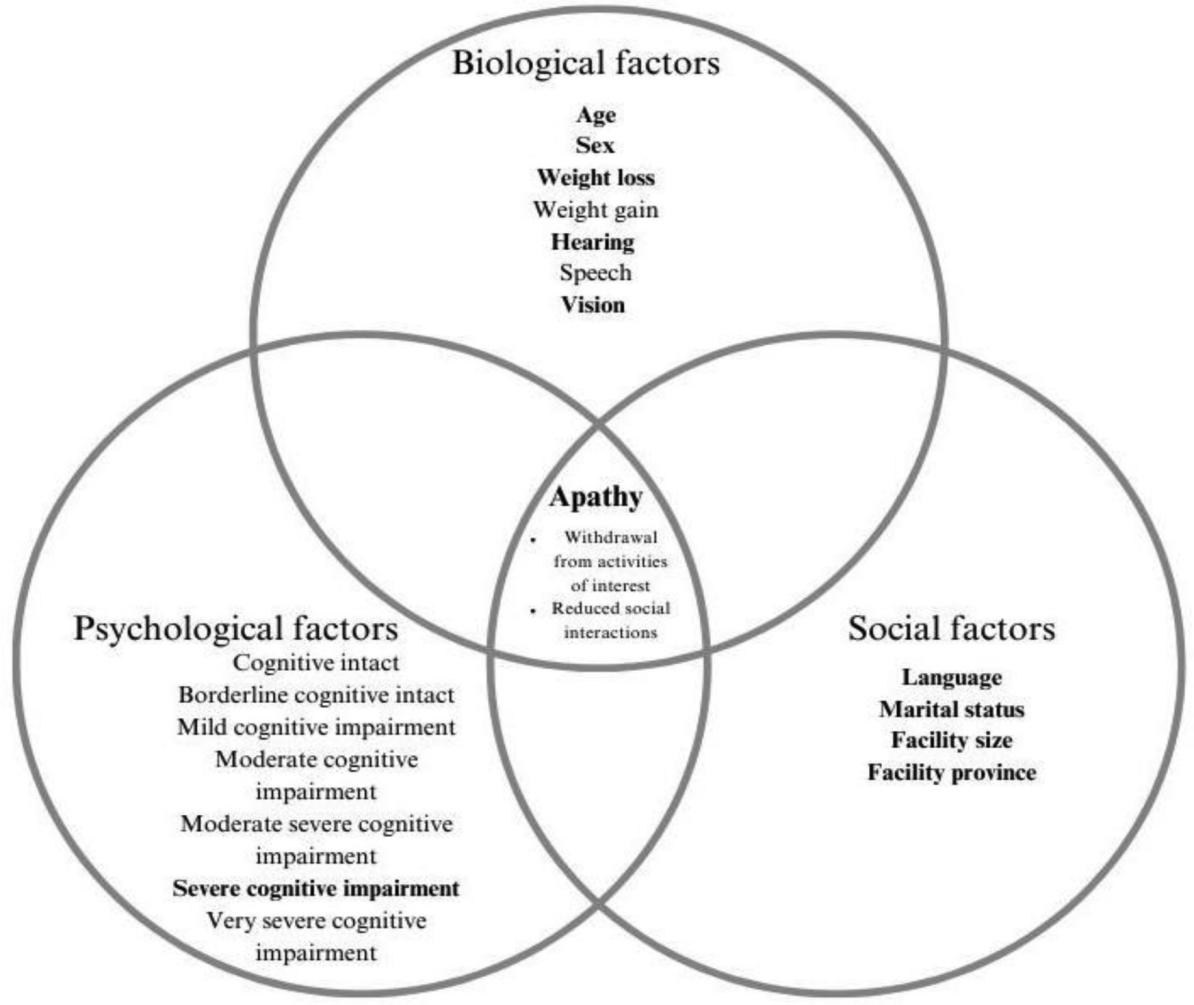
The biopsychosocial model of apathy in LTCF.

Figure 3 shows the theoretical framework for the regression results modelling the predictors of apathy among Canadian LTCF newly admitted residents. With regards to biological variables (Table 2), the predictors of apathy were age groups- less than 65 years of age (OR 1.26 (95% CI 1.16, 1.37), between 65 and 74 years of age (OR 1.30 (95% 1.22, 1.39) and between 75 and 84 years of age (OR 1.11 (95% CI 1.06, 1.16); weight loss (OR 1.49 (95% CI 1.38, 1.2); hearing- minimal difficulty (OR 1.13 (95% CI 1.08, 1.19), hears in special situations (OR 1.19 (95% CI 1.11, 1.26), and highly impaired (OR 1.62 (95% CI 1.41, 1.85); vision- minimal impaired (OR 1.17 (95% CI 1.12, 1.23), moderately impaired (OR 1.39 (95% CI 1.29, 1.50), and severely impaired (OR 1.14 (95% CI 1.11, 1.17). Concerning psychological variables (Table 2), severe cognitive impairment (OR 2.60 (95% CI 2.42,2.28) was the strongest predictor of apathy. With respect to social variables (Table 2), the predictors were French language (OR 1.27 (95% CI 1.15,1.42); unspecified marital status (OR 1.14 (95% CI 1.04, 1.24); medium sized facility (OR 1.18 (95% CI 1.14,1.22), and facility province (Saskatchewan) (OR 1.58 (95% CI 1.49–1.68).

**Table 2.** The biopsychosocial predictors of apathy among Canadian LTCFs residents, 2015–2019 fiscal year

Biological Variables	Adjusted odds ratio (95% Confidence limits)	*p values
Sex (Ref = female)		
Male	1.09 (1.04–1.13)	** < 0.0001
Other (e.g., hermaphrodite)	1.28 (0.60–2.721)	0.5179
Age group (Ref = 85 + years)		
Less than 65	1.26 (1.16–1.37)	** < 0.0001
65–74	1.30 (1.22–1.39)	** < 0.0001
75–84	1.11 (1.06–1.16)	** < 0.0001
Weight gain (Ref = No)	1.13 (0.97–1.32)	0.1312
Weight loss (Ref = No)	1.49 (1.38–1.62)	** < 0.0001
Hearing (Ref = Adequate)		
Minimal difficulty	1.13 (1.08–1.19)	** < 0.0001
Hears in special situations	1.19 (1.11–1.26)	** < 0.0001
Highly impaired	1.62 (1.41–1.85)	** < 0.0001
Uses speech to communicate (Ref = No)	0.93 (0.83–1.04)	0.1955
Vision (Ref = Adequate)		
Minimal impaired	1.17 (1.12–1.23)	** < 0.0001
Moderately impaired	1.39 (1.29–1.50)	** < 0.0001
Highly impaired	1.04 (0.94–1.15)	0.5081
Severely impaired	1.37 (1.18–1.59)	** < 0.0001
**Psychological variables (Ref = Intact)**		
Borderline Intact	1.51 (1.40–1.63)	** < 0.0001
Mild impairment	1.40 (1.30–1.49)	** < 0.0001
Moderate impairment	1.72 (1.62–1.84)	** < 0.0001
Moderate severe impairment	1.89 (1.75–2.05)	** < 0.0001
Severe impairment	2.60 (2.42–2.80)	** < 0.0001
Very severe impairment	2.34 (2.12–2.60)	** < 0.0001
**Social Variables**		
Primary language (Ref = Eng)		
French	1.27 (1.15–1.42)	** < 0.0001
Other	0.78 (0.74–0.82)	** < 0.0001
Marital status (Ref = Married)		
Never married/widowed/ separated/divorced	0.88 (0.81–0.96)	0.1107
Unspecified	1.14 (1.04–1.24)	**0.0054
Facility size (Ref = Large)		
Medium	1.18 (1.14–1.22)	** < 0.0001
Small	1.11 (0.10–1.23)	0.568
Facility Province (Ref = British Columbia)		
Manitoba	0.80 (0.74–0.93)	** < 0.0001
Newfoundland & Labrador	1.06 (0.98–1.16)	0.2384
Ontario	0.46 (0.44–0.48)	** < 0.0001
Saskatchewan	1.58 (1.49–1.68)	** < 0.0001
Yukon	0.33 (0.21–0.53)	** < 0.0001

*p value = logistic regression.

** = significant values.

## Discussion

This study aimed to investigate the prevalence and predictors of apathy among residents of Canadian LTCF. Apathy was present in all age groups and various disorders. The prevalence of apathy as measured by the Apathy Index of the MDS 2.0 was 12.5% among a large sample of the Canadian LTCF residents. This corroborates with previous studies, including a Dutch study involving 199 residents that reported a prevalent rate of 12% (Aalten et al., 2005), a UK study involving 1,419 participants utilizing the Neuropsychiatric Inventory to rate apathy that reported the prevalence of clinically significant apathy to be 21.4% (Sommerlad et al., 2022), and another Dutch study comprising of 290 lTCF residents from nine LTCF which found that the prevalence of apathy was 19% (Wetzels et al., 2010). However, the prevalence rate in the current study was substantially lower than the range of 19–88% as reported in a systematic review and meta-analysis of 25 studies (Zhu et al., 2019) and a study in the Netherlands which found a 28% prevalence of apathy among LTCF residents with stroke (van Almenkerk et al., 2015). The discrepancies in the prevalence rates across studies could be attributed to various factors, including differences in the demographic characteristics of the participants, settings of the studies, and methodologies employed in assessing apathy. In the current study, the measurement tool used to assess apathy focused only on two items: “withdrawal from activities of interest (e.g., no interest in long-standing activities or being with family, friends)” and “reduced social interaction” (e.g., less talkative, more isolated) (Morris et al., 2012, p.2) suggesting that the behavioral and social domains of apathy were captured while the emotional and cognitive domains of apathy were not accounted for. Nonetheless, in the absence of a comprehensive tool to measure apathy, the Apathy Index of the MDS 2.0 provides an important starting point to increasing awareness about apathy in the Canadian LTCF.

In relation to the prevalence of apathy among different age groups, the current study revealed that apathy was more frequent among residents who were younger than 65 years compared to those in the older age group (85 years and over). This is consistent with previous studies investigating the prevalence of neuropsychiatric symptoms in young-onset dementia (Bauhuis et al., 2020; Mulders et al., 2014; 2016), but in contrast to studies involving persons with advanced dementia (Selbaek et al., 2014; van Reekum et al., 2005; Van Vliet et al., 2013; Zuidema et al., 2009). It has been suggested that apathy is one of the primary reasons for institutionalization in individuals with young-onset dementia (Bakker et al., 2013). Early identification of apathy in this age group may be valuable for testing experimental medicines aimed at this syndrome or its associated symptoms (Dujardin et al., 2014).

Furthermore, apathy and depression frequently co-occur, as observed in over half (21.3% with moderate and 33.4% with high scores on DRS) of our sample. While some symptoms overlap between apathy and depression, each condition has distinct characteristics (Levy et al., 1998; Lueken et al., 2007; Starkstein et al., 2005): apathy is marked by blunted emotional response, indifference, low social engagement, diminished initiation, and poor persistence (Ishizaki & Mimura, 2011); depression, on the other hand, includes dysphoria, suicidal ideation, self-criticism, guilt feelings, pessimism, and hopelessness (Levy et al., 1998). Furthermore, apathy and depression differ in the brain regions implicated and have different progression patterns (Fahed & Steffens, 2021). This has implication for the use of anti-depressants in this population because treatment with selective serotonin reuptake inhibitor (SSRI) antidepressants may worsen apathy (Masdrakis et al., 2023). In our study, 14% of residents who exhibited apathy were treated with antidepressants.

Regarding the biological predictors of apathy, age emerged as a predictor, demonstrating that younger residents (<65 years) are more likely to be apathetic compared to older age groups. This finding aligns with the results of previous cross-sectional studies (Appelhof et al., 2019; Jao et al., 2018) and appears to be somewhat counterintuitive given that one might expect higher frequencies of apathy in the oldest age group, suggesting that younger residents in LTCF might experience factors that predispose them to apathy, such as early onset dementia or other comorbidities that necessitate long-term care at a younger age or it might be that younger individuals in LTCF might experience greater challenges in adjusting to the institutional environment, leading to higher levels of apathy (Van Malderen et al., 2013). The relatively high prevalence of apathy in the 65–74 and 75–84 age groups emphasizes the need for targeted interventions across different age categories.

In congruence with previous research, male sex was a predictor of apathy (Jao et al., 2020; Vilalta-Franch et al., 2013). This highlights the importance of personalized and sex/gender-sensitive approaches to enhancing the well-being of LTCF residents with apathy. In addition, weight emerged as an important variable, with weight loss associated a higher likelihood of apathy compared to those without. Volicer et al. (2013) noted a similar result, however, due to the cross-sectional nature of this study, it is unclear which came first, the weight change or the apathy and to which direction the temporal sequence may occur. Nonetheless, weight loss management through avoidance of certain medications that increases the risk of anorexia such as psychoactive medications and adequate treatment of medical disorders associated with anorexia including vit B12 deficiency and gastrointestinal disorders, may play a crucial role in mitigating the development of apathy among newly admitted residents in LTCF (Volicer et al., 2013).

Sensory impairments in hearing and vision also emerged as significant predictors. Various levels of hearing impairments (minimal, moderate, and high) were associated with a higher likelihood of apathy than adequate hearing. Similarly, various levels of visual impairment were associated with apathy, with moderately impaired vision showing the highest likelihood (adjusted odds ratio: 1.39; p < 0.0001). Speech impairment, indicated by an adjusted odds ratio of 1.14 (p < 0.0001), suggests that residents with speech impairment are more likely to exhibit apathy than those without. This could be attributed to the potential isolation, communication barriers, and resultant reduced social engagement experienced by individuals with speech impairment. König et al. (2019) highlighted the efficacy of using speech characteristics, including acoustic, semantic, and prosodic features, as reliable indicators of apathy among individuals with speech impairments. Notable changes in speech, such as flatter intonation and decreased volume, alongside reduced vocabulary, and varied content, mirror the emotional and motivational deficits typical of apathy. Prosodic features such as extended pauses and speech disruptions also indicate apathy-related cognitive and emotional disturbances (König et al., 2019). In LTCF, early identification of apathy among residents with speech impairments by integrating automatic speech analysis into routine assessments can enhance care and enable timely intervention. These findings suggest that interventions aimed at improving sensory function might mitigate apathy in this population.

In terms of psychological factors, this current study showed that cognitive performance, as measured by CPS, has a significant association with apathy among newly admitted Canadian LTCF residents. This association was stronger for all levels of cognitive impairment. The early recognition of high cognitive performance scores could be instrumental in implementing early interventions and tailoring care plans for residents in LTCF. More importantly, interventions aimed at stimulating and supporting executive functions may be beneficial in mitigating the progression of apathy (Drijgers et al., 2011). Additionally, considering that apathy is associated with poor initiation, structured, and guided activities that encourage participation and engagement without relying heavily on self-initiation might be effective in promoting social interaction and mental stimulation among LTCF residents (Drijgers et al., 2011). Evidence also suggests that the presence of apathy is a potential risk factor for conversion to dementia in people with mild cognitive impairment (Lanctôt et al., 2017; Robert et al., 2006; van Dalen et al., 2018). The progression from mild to severe cognitive impairment showing a graded increase in the odds of apathy underscores the importance of early cognitive interventions and continuous monitoring to manage apathy effectively.

Social factors play an important role in predicting apathy. In this study, language, particularly French, shows a statistically significant association with apathy, with adjusted odds ratio 1.27 (p < 0.0001), indicating that residents who speak this language are more likely to exhibit apathy compared to those speaking English language. This suggests that linguistic and possibly cultural factors may play a role in the display or recognition of apathy among LTCF residents. Further, our findings suggests that those who speak other languages are at lower odds of exhibiting apathy (adjusted odds ratio: 0.78; p < 0.0001). This underscores the importance of language concordance (Hsueh et al., 2021) in the recognition of apathy among speakers of other languages in LTCF. It would be insightful to explore whether these associations are influenced by factors such as communication barriers, social integration, cultural perceptions of mental health, and emotional expression. Additionally, marital status impacts apathy, with those of unspecified marital status showing higher odds compared to residents who were married. This may point to the complexities of social support systems and their influence on residents’ health outcomes.

Facility size also emerged as a crucial predictor, with medium sized facility associated with a higher likelihood of apathy (adjusted odds ratio 1.18, p < 0.0001) compared to large sized facility, which might be due to differences in the availability of resources and activities (Mansbach et al., 2016). The relationship between the institutional environment and apathy is well documented (Chaudhury et al., 2017; Jao et al., 2015, 2019). This finding also aligns with the broader literature highlighting the protective effects of personalized care on apathy (Zuidema et al., 2010). Smaller facilities may facilitate more personalized care due to a lower staff-to-resident ratio (Zuidema et al., 2010). This personalized attention can be pivotal in recognizing and addressing early signs of apathy. In contrast, medium and larger facilities, despite having more resources, might struggle with providing individualized care to the same extent.

Furthermore, the province of the facilities emerged as a notable predictor of apathy, with residents in Manitoba, Ontario, and Yukon showing statistically significant lower likelihood of apathy while those in Saskatchewan demonstrating a higher likelihood of apathy compared to residents in British Columbia. This finding highlights the importance of considering geographical differences when developing interventions for apathy rather than just focusing on individual and institutional factors because geographical variation could be influenced by various factors, such as differences in healthcare policies, and socio-cultural contexts across provinces. This finding warrants further exploration to better understand the underlying mechanisms.

## Implication for practice/policy

In this study, apathy was examined from a biopsychosocial perspective, and the results of our findings showed that various biological, psychological, and social factors are associated with apathy among residents in LTCF. To this end, health care professionals and policymakers should consider the following:

- Create an awareness campaign about apathy at the institutional, provincial, and national levels.
- Develop a standard assessment tool for evaluating apathy in LTCF that considers the needs and preferences of younger people.
- Screen for apathy in all residents presenting with high cognitive performance scores using both systematic speech analysis and apathy screening tools to enhance early detection of apathy.
- Incorporate technology-based meaningful activities into existing programs to keep residents engaged during the day while simultaneously preventing additional workloads for care staff.

## Strength and limitation of the study

This study has several strengths, including the use of a large population sample, which is representative of the LTCF population. This enhances the generalizability of the findings across Canadian populations and provides a solid cross-section of apathy within the LTCF. Additionally, the results indicated that the prevalence of apathy among Canadian LTCF residents is comparable to existing studies, signifying the utility of the apathy index in the early detection of apathy among this population. This study also provides a thorough and multifaceted exploration of predictors of apathy, encompassing biological, psychological, and social factors, and offers a holistic view of the factors influencing apathy among LTCF residents. While this study was the first to investigate the prevalence of apathy among a very large sample of residents across Canadian LTCF, using data from the MDS 2.0, a validated tool approved for use in all healthcare settings across Canada, not all provinces and territories are included. This suggests that caution should be exercised when generalizing the results to all the provinces and territories in Canada. Furthermore, the use of this data, which is not primarily for clinical diagnosis or research purposes, might have made it difficult to examine more nuanced relationships between apathy and the various variables included in this study. Future studies should adopt a more rigorous methodology to examine these relationships.

## Conclusion

This study examined the prevalence and predictors of apathy among residents of Canadian LTCF. The study found a complex interaction between apathy and various biological, psychological, and social factors. Apathy was found to be associated with biological indicators such as age, weight loss, and sensory deficits, highlighting the complicated relationship between apathy and physical health of LTCF residents. Psychological factors, particularly cognitive impairment, have a complex relationship with apathy. Severe cognitive impairment was associated with the highest risk for apathy than very severe impairment. This indicates that cognitive function and apathy are not linearly related. The study also found that marital status, facility size, and facility province were significantly associated with apathy. This highlights the impact of social settings and relationships on LTCF residents’ well-being. Apathy was also predicted by language, underscoring the importance of efficient communication and meaningful social interactions for preventing apathy.

Given these findings, a comprehensive and individual-focused approach to addressing and reducing LTCF apathy LTCF is essential. Interventions must address the physical, social, and psychological well-being of residents with apathy. Future research should examine the qualitative aspects of social connections and activity engagement, as well as their interactions with biological and psychological parameters, to better understand how they affect apathy in LTCF residents. This may help to develop approaches and interventions to improve the quality of life and well-being of LTCF residents. It is important to note that facility province was a strong predictor of apathy, and to the best of the author's knowledge, no research has explored this factor. Future studies should expand this factor and explore more effective interventions to mitigate apathy at the provincial level.
